# Aspects of the development of *Ixodes anatis* under different environmental conditions in the laboratory and in the field

**DOI:** 10.1186/s13071-021-04601-z

**Published:** 2021-01-28

**Authors:** Natasha Bansal, William E. Pomroy, Allen C. G. Heath, Isabel Castro

**Affiliations:** 1 School of Agriculture and Environment, Massey University, Private Bag 11222, Palmerston North, New Zealand; 2 Wildbase Research, Massey University, Private Bag 11222, Palmerston North, New Zealand; 3 School of Veterinary Science, Massey University, Private Bag 11222, Palmerston North, New Zealand; 4 AgResearch Ltd, Hopkirk Research Institute, Massey University, Private Bag 11008, Palmerston North, 4442 New Zealand

**Keywords:** Life-cycle, Kiwi tick, Endophilic, *Ixodes anatis*

## Abstract

**Background:**

Numerous laboratory and fewer field-based studies have found that ixodid ticks develop more quickly and survive better at temperatures between 18 °C and 26 °C and relative humidity (RH) between 75 and 94%. *Ixodes anatis* Chilton, 1904, is an endophilic, nidicolous species endemic to North Island brown kiwi (*Apteryx mantelli*) (NIBK) and the tokoeka (*Apteryx australis*), and little is known about the environmental conditions required for its development. The aims of this study were to determine and compare the conditions of temperature and RH that ensure the best survival of the kiwi tick and the shortest interstadial periods, in laboratory conditions and outdoors inside artificial kiwi burrows.

**Methods:**

Free-walking engorged ticks were collected off wild kiwi hosts and placed in the laboratory under various fixed temperature and humidity regimes. In addition, sets of the collected ticks at different developmental stages were placed in artificial kiwi burrows. In both settings, we recorded the times taken for the ticks to moult to the next stage.

**Results:**

Larvae and nymphs both showed optimum development at between 10 °C and 20 °C, which is lower than the optimum temperature for development in many other species of ixodid ticks. However, larvae moulted quicker and survived better when saturation deficits were < 1–2 mmHg (RH > 94%); in comparison, the optimum saturation deficits for nymph development were 1–10 mmHg.

**Conclusions:**

Our results suggest that the kiwi tick has adapted to the stable, but relatively cool and humid conditions in kiwi burrows, reflecting the evolutionary consequences of its association with the kiwi.
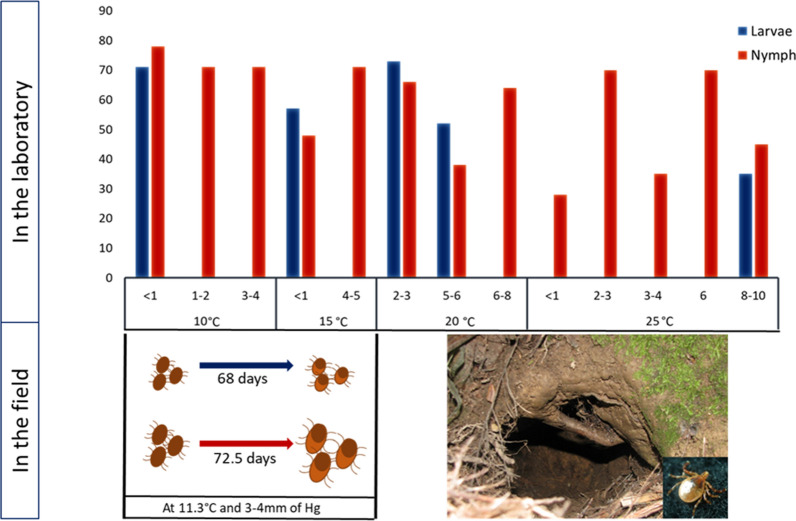

## Introduction

The amount of time that each life-cycle stage of ticks takes to complete is determined by interactions between temperature and moisture (relative humidity [RH]) in the off-host habitat [1–3]. During protracted off-host (questing) and engorged periods of their life, ticks are more prone to desiccation [4], and their ability to perform bodily functions largely depends on water vapour absorption [5]. Thus, optimum developmental conditions ensure faster progress to the next stage of the life-cycle and better chances of survival. Numerous laboratory-based studies have explored the response of different species of ixodid ticks to microclimates and the influence of these on developmental times [6–11]. The results of most of these studies are similar in showing that, for optimum development, ixodid ticks prefer temperatures of between 18 °C and 26 °C and RH of between 75 and 94%. Some of these studies showed that an increase in temperature within the preferred range reduced moulting times, and that although some species were able to tolerate temperatures up to 38 °C, mortality rates increased at these higher temperatures. At lower temperatures, such as 4–8 °C, some species continue to develop, but at a greatly reduced rate and with higher mortality. Similar results have been demonstrated in the small number of field studies that have been conducted with various tick species [12–16].

*Ixodes anatis* Chilton, 1904, is a host-specific ixodid tick found on apterygid birds, which include the North Island brown kiwi (*Apteryx mantelli*) (NIBK) and the tokoeka (*Apteryx australis*); as such, this tick is endemic to New Zealand [17, 18]. It is an endophilic, nidicolous species which has only been recovered either from the body of the hosts or within their burrows. *Ixodes anatis* of all stages are prevalent in kiwi burrows at some sites throughout the year [19, 20].

Our aims in this study were two-fold: to determine in the laboratory the conditions of temperature and RH that ensure the best survival and shortest interstadial periods for the kiwi tick, and to contrast these with those of ticks of different stages placed in artificial kiwi burrows outdoors. To date, little is known about the environmental conditions that are ideal for the development of *I. anatis* and therefore our null hypothesis was that this species would behave comparably to other species with similar ecological requirements, such as *Ixodes uriae, I. arboricola* and *I. trianguliceps* [21] or species in other genera, such as *Amblyomma* and *Archaeocroton* [21, 22], all of which are examples of nidicoles.

## Materials and methods

### Experimental design

**Fig. 1:**
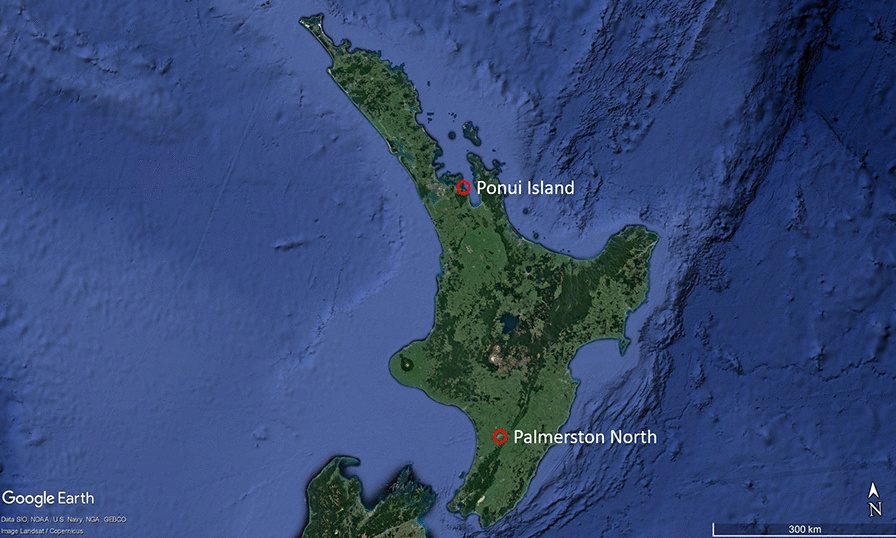
Map showing the two sites (red circles) used in experiments designed to find the best temperature and humidity conditions for the development of *Ixodes anatis*, the kiwi tick. Ponui Island is where the ticks were collected, and Massey University in Palmerston North is where both the field experiment and laboratory experiments were conducted

Two series of experiments were conducted to determine the optimum developmental conditions for *I. anatis*. In the first, engorged larvae, nymphs and adults were incubated under laboratory conditions (laboratory experiments); in the second, engorged ticks were maintained in artificial kiwi burrows (field experiments) in a forested area close to the laboratory (40.3709°S, 175.6303°E; Fig. 1). In all experiments, the pre-moult period was defined as the time from when a fully engorged tick was placed in the incubator or burrow to the time it started moulting. Moulting duration was the time from when the tick started moulting until the time the new stage first appeared. Moulting success was the proportion of ticks that were able to successfully ecdyse. For females, preoviposition was the time from the moment the female was placed in the incubator to the time it started laying eggs, and oviposition was the time taken for the female from the start to stop of egg laying.

### Tick collection

Ticks were collected from NIBK inhabiting a high-density population of one bird per hectare on Ponui Island (Inner Hauraki Gulf, New Zealand; 36.8622°S, 175.1842°E; Fig. 1) [23]. These birds had been observed to have high densities of ticks, with up to 250 individuals recorded on one host [18, 24]. Between April and June 2016 (for the laboratory experiments) and March 2018 (for the field experiment), detached, free-walking engorged ticks were collected. No ticks were forced off the hosts; those collected were found walking on their feathers, on the surface of their bodies, on bird handlers and inside the bags used to cover the birds during handling. We assumed these ticks would have been naturally leaving the hosts after being satiated. All ticks used also looked fully engorged to the eye. Ticks were separated into the three stadial groups (larva, nymph, adult female), placed in plastic containers with fresh vegetation to provide moisture and stored at 4 °C, for a mean duration of 5 days (± 5 days), until they arrived in the laboratory at Massey University, Palmerston North (546 km distant from the study site; Fig. 1).

### Tick identification

Only two species of ticks have been found from kiwi at the study site, namely *Haemaphysalis longicornis* and *I. anatis*, and as New Zealand has only one species of *Haemaphysalis* present, that genus is readily separated from the genus *Ixodes* based on palpal morphology. This and other features separating the species of *Ixodes* in New Zealand (shape of scutum, presence or absence of coxal spurs, etc.) were understood by the authors and taken into consideration when ticks were collected and identified, using the keys in Dumbleton [17]. Only those ticks identified as *I. anatis* were used in this study.

### Pilot experiment

To test the combined effect of temperature and humidity on the stages of the tick we needed to establish conditions of different RHs and maintain these at different temperatures. Winston and Bates [25] developed protocols to create various RHs for exactly this purpose by dissolving enough solid salt to super saturate distilled water at boiling point. The basic principal of this mechanism is that any saturated salt solution, when placed at a constant temperature produces a fixed vapour pressure which is in equilibrium with the vapour pressure of water and thus expresses a fixed RH [25, 26]. We conducted a pilot test using salt solutions from their protocols [25, 26], with the expressed purpose of being able to produce a range of RHs for further experiments with the collected ticks. The salt solutions we used to achieve the required RH are given in Table 1. These solutions were placed at the bottom of sealed plastic containers with mesh lids, and an iButton Hygrochron™ Temperature/Humidity Loggers (model DS1923; Maxim Integrated, San Jose, CA, USA) was hung from the lid so it was at the same level as the ticks. The entire setup was placed in fixed temperature incubators at the selected temperatures of 5 °C, 10 °C, 15 °C, 20 °C, 25 °C and 30 °C. The hygrometers were set to record temperature and humidity every 10 min for 1 week. Despite numerous attempts, not all the salt solutions produced the desired RHs reported in Winston and Bates [25] and therefore we used the actual RHs achieved (Table 1) as our final RHs for the main laboratory experiment.

**Table 1:** List of saturated solutions used at different temperatures to achieve the required relative humidity

Saturated salt solutions	Relative humidity^a^											
	5 °C		10 °C		15 °C		20 °C		25 °C		30 °C	
	Expected	Actual	Expected	Actual	Expected	Actual	Expected	Actual	Expected	Actual	Expected	Actual
Sodium chloride + potassium chloride							70	64.9 ± 0.61			70	66.8 ± 0.8)
Sodium chloride	75	77.1 ± 0.62	75	62.1 ± 0.47	75	66.8 ± 1.18	75	63.3 ± 0.58	75	73.9 ± 0.56	75	72.9 ± 0.6
Potassium bromide			85	83.4 ± 0.48			85	66.3 ± 0.71	85	63.9 ± 0.71		
Potassium chloride									85	83.6 ± 0.98	85	84.6 ± 0.17
Potassium nitrate	95	92.4 ± 1.86	95	95.5 (± 0.86)	95	93.9 ± 0.1.53			90	87.8 ± 1.2	90	81.6 ± 2.01
Potassium sulphate	99	64 .7 ± 0.50	99	96.4 ± 0.45	99	96.4 ± 2.56	99	85.6 ± 1.58	95	67.5 ± 1.82	95	72.9 ± 1.60
Potassium dichromate									99	97.6 ± 0.23	99	98.8 ± 1.80
Sodium nitrite							65		65		65	

^a^Values for actual relative humidity (RH) are presented as the mean ± standard deviation

### Laboratory experiment—effects of a range of temperature and humidity conditions

Engorged larvae and nymphs were individually placed into small fabric mesh pockets that were suspended above the saturated salt solutions (Table 1). These were then incubated at a range of temperatures (5 °C, 10 °C, 15 °C, 20 °C, 25 °C and 30 °C). Twenty engorged larvae and ten engorged nymphs were used at each humidity and temperature combination. In addition, to measure preoviposition and oviposition time, we divided 12 engorged adult females into four groups of three and then incubated two of these groups at 15 °C and 93% and 96% RH, respectively; one group at 10 °C and 94% RH; and one at 20 °C and 85.5% RH. Eggs obtained from these female ticks were subsequently divided into batches and placed in mesh bags (50 eggs/bag) at all temperature and RH combinations (Tables 1, 2). Temperature and RH were measured every hour using iButton Hygrochron™ Temperature/Humidity Loggers. The ticks were observed every 2 days for evidence of development, for a total of 6 months. In this experiment the hypothesis was that larvae, nymphs and females of *I. anatis* would have more successful and faster developmental times at temperatures between 15 and 20 °C and RH > 90% than in conditions outside this range.

**Table 2:** Survival and developmental time of engorged larvae and nymphs of *Ixodes anatis* under the tested laboratory conditions

Temperature (°C)	RH (%)	Saturation deficit (mmHg)	Larvae				Nymphs			
			Premoult^a^		Moulting^b^		Premoult^a^		Moulting^b^	
			Time (days)	Survival %	Time (days)	Survival %	Time (days)	Survival %	Time (days)	Survival %
5	64 ± 0.50	1–2		0		0		0		0
	77 ± 0.62	1–2		0		0		0		0
	92 ± 1.86	< 1		0		0		0		0
10	62 ± 0.47	3–4		0		0	71 ± 1.70	100	6.3 ± 1.7	100
	83 ± 0.48	1–2		0		0	71 ± 0.84	100	7.4 ± 0.8	100
	94 ± 0.45	< 1	64 ± 4.39	100	9.8 ± 3.7	100	78 ± 0.84	100	6.6 ± 0.8	100
	95 ± 0.86	< 1	78 ± 1.23	100	18.3 ± 5.02	100	78 ± 1.33	100	7 ± 1.3	100
15	67 ± 1.18	4–5		0		0	71	100	7	30
	94 ± 0.1.53	< 1	57 ± 1.41	100	14.9 ± 1.4	100	50 ± 0.95	100	6.7 ± 0.9	100
	96 ± 2.56	< 1	57 ± 1.33	100	14.75 ± 1.3	100	36 ± 0.84	100	7.4 ± 0.8	100
20	63 ± 0.58	6–8		0		0	62 ± 1.51	100		0
	65 ± 0.61	6–8		0		0	66 ± 1.03	100	9	20
	66 ± 0.71	5–6	52 ± 0.67	100	14.15 ± 0.7	100	38 ± 0	100	12.6 ± 2.95	100
	86 ± 1.58	2–3	73 ± 0.82	100	6.6 ± 0.8	100	66 ± 1.93	100	14 ± 4.8	100
25	64 ± 0.71	8–10	35	60	7	60	35 ± 15.65	50	8.8 ± 2.5	10
	67 ± 1.82	8		0		0	56	10		0
	74 ± 0.56	6		0		0	70	100		0
	84 ± 0.98	3–4		0		0	35 ± 1.64	50	7	30
	88 ± 1.2	3		0		0	70 ± 2.54	90	9 ± 1.7	90
	98 ± 0.23	< 1		0		0	28	70	7	40
30	67 ± 0.87	10–12		0		0		0		0
	73 ± 1.60	8–10		0		0		0		0
	73 ± 0.6	8–10		0		0		0		0
	82 ± 2.01	5–6		0		0		0		0
	85 ± 0.17	4–5		0		0		0		0
	99 ± 1.80	< 1		0		0		0		0

Values in table are presented as the mean ± standard deviation

There were 20 larvae and 10 nymphs at each temperature and RH combination

The saturation deficit of air in each chamber was calculated using the formula from Randolph and Storey [27]

^a^ Pre-moult refers to the number of ticks that survived and started moulting

^b^Moulting refers to the actual time of moulting from attachment to complete emergence

### Field experiment

**Fig. 2:**
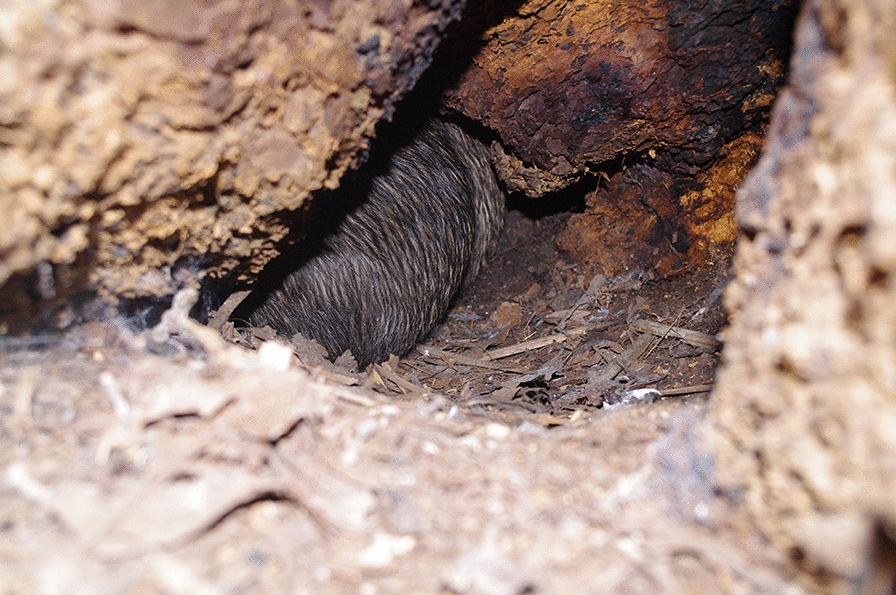
Example of a kiwi burrow (with a kiwi in it), which was a model for the burrows dug for the field studies at Massey University

Engorged larvae and nymphs were placed in artificial burrows (*n* = 12) from June to August (Southern Hemisphere winter) 2018. At Massey University, horizontal burrows were dug in a forest environment consisting of clay/silt loam-type soil, with the aim to imitate natural burrows. These simulated burrows were approximately 120–150 mm in diameter and 600 mm deep (Fig. 2). A larger chamber was constructed at the end to mimic a typical kiwi-constructed burrow (D. Vieco-Galvez, unpublished data). Ten nymphs and 20 larvae were placed in each burrow in mesh pockets (one for each stage). These ticks were checked every 2 to 3 days to record moulting. Temperature and RH were recorded every hour using iButton Hygrochron™ Temperature/Humidity Loggers. For this experiment, we expected both the stages to follow the same pattern as found in the laboratory experiment.

### Statistical analysis

One-way analysis of variance (ANOVA) was carried out in R version 2013 (http://www.R-project.org/) to test for significance between the number of days taken to start and complete moult for the different stages, where applicable. Linear regressions were also performed to test the significance of temperature and RH on preoviposition and oviposition times in females.

The saturation deficit (SD), which is the amount of water vapour required to saturate air (in mmHg) was calculated using the formula: SD = (1 − RH/100) × 4.9463e^0.0621T^ (where RH is relative humidity in  %, e is the mathematical constant ‘Euler’s number’ and T is temperature in  °C) [27]. For both the laboratory experiment and the field experiment, results were reported using both RH and the corresponding SD at the given temperatures.

## Results

### Laboratory experiment

Percentage survival of larvae and nymphs and the duration range of pre-moult are summarised in Table 2. None of the larvae or nymphs showed signs of development at 5 °C, even after 120 days of observation, and all ticks placed at 30 °C died within 20 days regardless of environmental humidity. Both larvae and nymphs survived at temperatures between 10 and 20 °C, with nymphs tolerating a wider range of temperatures than larvae. The greatest overall survival and shortest moulting times for both larvae and nymphs happened at 10 °C and 94–95% RH, representing a SD < 1 mmHg. Larvae at > 5 mmHg SD did not survive, nor did nymphs at > 10 mmHg SD.

The larvae only moulted at RH > 94% at 10 °C, with a mean pre-moult period of 60 (range 64–80) day, and the mean cumulative time for all larvae to complete a moult was 14 (range 5–21) days (Table 2). At an RH > 93% at 15 °C, the mean premoult period for larvae was 56 (range 54–57) days and the mean moulting duration was 15 (range 14–17) days. At 20 °C and between 2 and 6 mmHg SD, larvae had a mean premoult period of 73 (range 73–75) days, compared to a mean of 52 days at 5–6 mmHg, which was a statistically significant difference (ANOVA:* F*_(1,44)_ = 4.061, *P* < 0.01). At 25 °C and 64% RH (approx. 8–10 mmHg SD), only 60% of larvae showed signs of development, with a 35-day premoult period and 7-day moulting duration. Larvae at all other experimental temperatures and RHs did not develop.

The nymphs were more tolerant to a greater range of temperature and RH than were larvae. Overall, a variable proportion of nymphs started premoult at each RH but only a small number completed the process (Table 2). At SD of > 3 mmHg, the nymphs showed evidence of fungal growth. All nymphs at 10 °C started premoult with a mean of 75 (range 69–80) days and completed moulting with a mean of 7 (range 4–9) days irrespective of RH (Table 2). At 15 °C, the premoult period for nymphs at 67% RH (SD approx. 4–5 mmHg) was 71 days, but only 30% of these completed moulting over a 7-day period. However, at 15 °C and a SD of < 1 mmHg, nymphs took significantly less time to moult (ANOVA: *F*_(1,28)_ = 4.19, *P* < 0.01) (Table 2). At 20 °C and an SD of between 6 and 8 mmHg, all nymphs started premoult but only 20% successfully completed the process, which they did over 9 days (Table 2). At 66% RH (SD between 5 and 6 mmHg), all the nymphs moulted, with a mean moult time of 14 days, but with a large variation in time (range 7–42 days). At 86% RH (SD between 2 and 3 mmHg), all nymphs had a premoult period of 38 days, taking 13 (range 7–14) days to complete the moult. At 25 °C and 88% RH (3 mmHg SD), 90% completed a moult.

All six female ticks at 15 °C and 93.86% RH and the three female ticks at 20 °C and 85.55% RH laid around 600–750 eggs each. Only one of the three ticks placed at 10 °C and only two of three placed at 15 °C and 96.37% RH laid eggs. As the temperature increased, the preoviposition period significantly decreased (*F* stat = 196, *df* = 2, *P* = 0.005) while the RH had no significant effect (Table 3). The remaining females did not lay eggs and died within 60 days of being placed in the incubator. No eggs hatched under any of the experimental conditions.

**Table 3:** Developmental times for the 12 female engorged ticks (three at each chamber) at given temperature and relative humidity regimes

Temperature (°C)	RH (%)	Saturation deficit (mmHg)	Preoviposition period (days)	Oviposition period (days)	Success rate (%)
10	94	< 1	33	7	33.3
15	94	< 1	27	9	100.0
15	96	< 1	27	9	66.7
20	86	2–3	19	9	100.0

### Field experiment

**Fig. 3:**
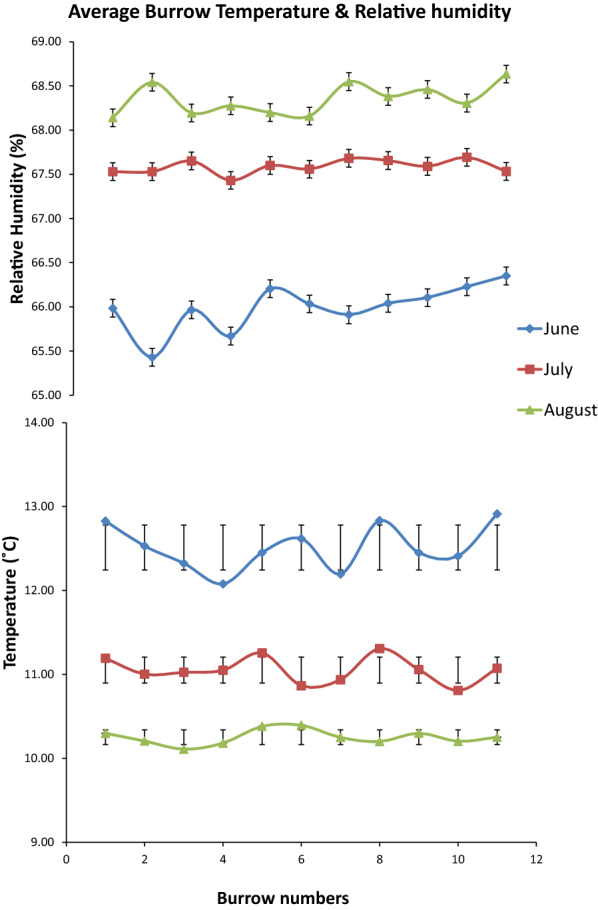
Average temperature and relative humidity (± standard error) in artificial kiwi burrows during June (blue), July (red) and August (green), 2018

**Fig. 4:**
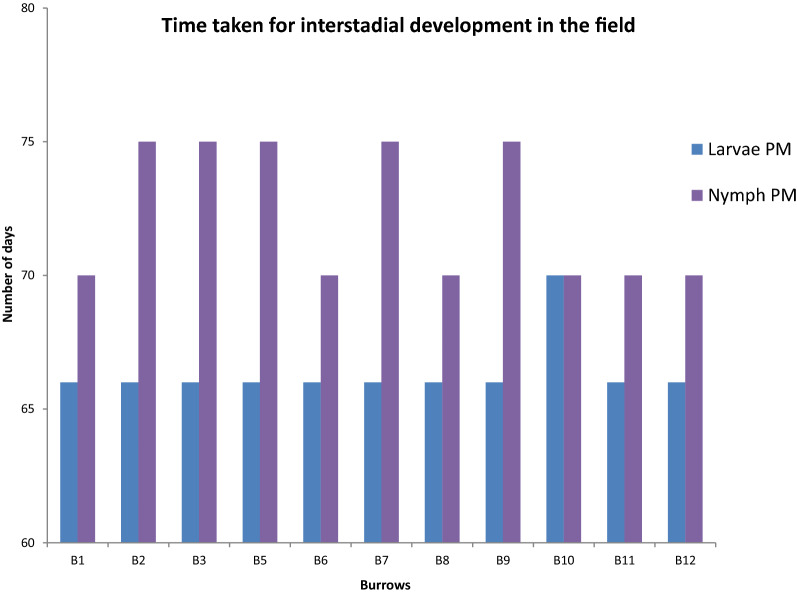
Time taken (in days) for development of immature stages of *Ixodes anatis* in the field experiments.* PM* Pre-moult duration

Of the 12 burrows, only 11 were included in the analysis because burrow 4 collapsed 16 days into the experiment. The overall mean temperature over the 3 months of the experiments was 11 °C (range 10–13 °C) and mean RH was 67% (range 65–69%). The mean (± standard deviation) temperature over all burrows was 13 °C ± 0.27 °C for June, 11 °C ± 0.15 °C for July and 10 ± 0.09 °C for August. The mean RH over all the burrows was 66 ± 0.26% for June 68 ± 0.08% for July and 68 ± 0.17% for August (Fig. 3). The corresponding SD of all the burrows ranged between 3 and 4 mmHg. Of the 220 engorged larvae placed in the burrows, 218 (99.1%) moulted to nymphs. Of the 110 engorged nymphs, 101 (91.8%) survived to moult; the remaining nine died after 40 days without completing development. Larvae in ten of the 11 burrows had a premoult period of 66 days and took 7 days to complete the moult. In burrow 10, the larval premoult period was 70 days with an overall duration of 5 days. Nymphs in six burrows had a 70-day pre-moult period with 75 days for the remainder. All nymphs with the exception of those in burrows 3 and 5 finished moulting in 8 days. Nymphs took 8 days to moult, with the exception of those in burrows 3 and 5 which took 6 days (Fig. 4).

## Discussion

Our experiment did not support our initial hypothesis that *I. anatis* would act comparably to most other nidicolous tick species in terms of conditions of preferred temperature and humidity.

Under laboratory conditions, the requirements for larval development were narrower than those for nymph development. Engorged larvae showed optimum development (moulting times and survival) at 10–20 °C when the SD was < 1–2 mmHg (RH > 94%). Engorged nymphs survived and moulted at temperatures up to 25 °C but, like larvae, appeared to favour a range of 10–20 °C, although they did have the ability to survive a somewhat drier atmosphere, tolerating a SD range of 1–10 mmHg. Unfortunately, the lack of a set of constant RH at each temperature prevented us from comparing the different conditions, which would have helped us discern the ideal range of conditions required for the *I. anatis* life-cycle. Females laid eggs at all temperatures and over the range of humidities tested, although the preoviposition period was from 6 to 14 days longer at SD < 1 mmHg than at SD 2–3 mmHg. However, due to the very small number of females tested, this result serves only as a loose guideline and needs to be further explored. The failure of eggs to hatch under the different temperature and RH combinations could be due to a number of reasons. The prolongation in duration of oviposition at the lower temperature may have exposed the eggs to a greater decline in their water balance than would have occurred at higher temperatures. Also, breaking the eggs into smaller batches may have possibly increased the surface area of the egg clumps and subjected them to increased dehydration. However, these possibilities require further investigation.

Under field conditions, the temperatures in the burrows varied only slightly across the 3 months of the study, remaining at the lower end of the favourable range for both larvae and nymphs found in the laboratory experiment. However, the RH was measured in the burrow air, not at the soil surface, which may have been slightly more humid. Previous experiments conducted on burrows (D. Vieco-Galvez, personal communication, 2018) have shown that while the external temperature fluctuates, the diurnal temperature within the burrow remains relatively constant, although the burrows do show a seasonality over the year. Moreover, over the year, the microclimate in a burrow is not as extreme as in the external environment and remains within a range of ± 6 units for both temperature and humidity.

In the burrows, the developmental success rate for both larvae and nymphs was very high (99 and 91.8%, respectively). In the laboratory, however, larvae exposed to similar conditions as those in the field (10 °C and 62.1–83% RH; SD 1–4 mmHg) did not survive. It is possible that the engorged larvae in burrows were in closer contact to available soil moisture and able to absorb it in through the cuticle or they experienced reduced water loss. Larvae in laboratory chambers were surrounded by humid atmospheric air, but at a level perhaps less than that experienced by the larvae in burrows. Ogden et al. [15] reported that even small fluctuations or changes in temperature and humidity can affect the developmental times in ticks. Therefore, it is also possible that these differences between the laboratory and field results may have been caused by our routine checks as larvae are less tolerant to minor changes in temperature and RH [28]. In addition, another study by Padgett and Lane [29] found that when larvae were left undisturbed, they had a higher success of moulting that those that were disturbed more often. For example, some ticks observed in the laboratory would have been exposed to more severe changes of temperature and humidity when extracted to assess survival than those in the field as some of the former were kept at considerably higher or lower temperatures than the general laboratory environment.

In previous studies with kiwi-occupied burrows [19, 20], larvae were most prevalent from January to June (summer and autumn) and lowest in October (spring; usually a damper season). Nymphs, on the other hand, were less prevalent in January, with the highest numbers reported from June to December. In the present study the artificial ‘burrows’ did not have any kiwi, which is very likely to have influenced temperature and humidity levels, as there would be no effect of physiological exhalations, body warmth [30] and deposited waste material.

In general, in many species of Ixodidae, immature stages survive better at moderate to high RH (> 90%) and between 18 °C and 25 °C but die off rapidly if the RH declines to 75% at these same temperatures [16, 29, 31, 32]. However, as in other species, the bioclimatic requirements of larvae are at the lower end of the range tolerated by the species overall. To a large extent this determines both the seasonal patterns and habitat suitability for the species because if larvae are disadvantaged, the life-cycle can be disrupted. Nymphs, however, are generally more desiccation resistant and have a better tolerance of higher temperatures than do larvae, with engorged females capable of withstanding even greater bioclimatic extremes [8, 28, 32, 33].

The kiwi is a nocturnal animal and can range widely in search of food as well as use a multitude of burrows within its range [34, 35]. Despite the original example of this species of tick being found on a duck, the kiwi tick is almost exclusively host specific and aberrant hosts are very rare 18], suggesting that it would be an advantage for the tick to be sedentary and to be capable of sustained quiescence in the event of the spasmodic presence of hosts.

The best survival strategy for the kiwi tick is to have a mix of stages in each burrow, ready to take advantage of the return of a host. A quicker development cycle for engorged larvae over the warmer time of year results in unfed nymphs that are able not only to withstand cooler times of the year but also the attendant added risks of dehydration. Unfed stages were not tested in these experiments, and such a study would throw additional light on the biology of *I. anatis* in relation to its host.

## Conclusion

In this study, the optimum temperature preferred by *I. anatis* to complete development was between 10 °C and 15 °C which is lower than that of many other species of ixodid ticks. We suggest that extended developmental times as a function of a preference for low temperature may be an adaptation for survival in burrows which are unoccupied for long periods as well as to the cold temperatures in New Zealand. There has been no success in finding questing *I. anatis* outside of kiwi burrows, reinforcing the inference of the tick’s sedentary nature and thus its adaptation to stable, but relatively cool and damp conditions in the burrows and reflecting the findings in this study as well as the evolutionary consequences of its association with the kiwi.

## Data Availability

The datasets used and/or analysed during the current study are available from the corresponding author on reasonable request.
